# The HMGB1-RAGE axis modulates the growth of autophagy-deficient hepatic tumors

**DOI:** 10.1038/s41419-020-2536-7

**Published:** 2020-05-07

**Authors:** Bilon Khambu, Honghai Hong, Sheng Liu, Gang Liu, Xiaoyun Chen, Zheng Dong, Jun Wan, Xiao-Ming Yin

**Affiliations:** 1 Department of Pathology & Laboratory Medicine, Indiana University School of Medicine, Indianapolis, IN USA; 2 Department of Pathology & Laboratory Medicine, Tulane University School of Medicine, New Orleans, LA USA; 3 The third afflilated hospital of Guangzhou Medical University, Guangzhou, Guangdong, China; 4 Department of Medical and Molecular Genetics, Indiana University School of Medicine, Indianapolis, IN USA; 5 Center for Computational Biology and Bioinformatics, Indiana University School of Medicine, Indianapolis, IN USA; 6 Department of Cellular Biology and Anatomy, Medical College of Georgia at Augusta University and Charlie Norwood VA Medical Center, Augusta, GA USA; 7 School of Informatics and Computing, Indiana University—Purdue University at Indianapolis, Indianapolis, IN 46202 USA

**Keywords:** Macroautophagy, Oncogenesis

## Abstract

Autophagy is an intracellular lysosomal degradative pathway important for tumor surveillance. Autophagy deficiency can lead to tumorigenesis. Autophagy is also known to be important for the aggressive growth of tumors, yet the mechanism that sustains the growth of autophagy-deficient tumors is not unclear. We previously reported that progression of hepatic tumors developed in autophagy-deficient livers required high mobility group box 1 (HMGB1), which was released from autophagy-deficient hepatocytes. In this study we examined the pathological features of the hepatic tumors and the mechanism of HMGB1-mediated tumorigenesis. We found that in liver-specific autophagy-deficient (*Atg7*^Δ*Hep*^) mice the tumors cells were still deficient in autophagy and could also release HMGB1. Histological analysis using cell-specific markers suggested that fibroblast and ductular cells were present only outside the tumor whereas macrophages were present both inside and outside the tumor. Genetic deletion of *Hmgb1* or one of its receptors, receptor for advanced glycated end product (*Rage*), retarded liver tumor development. HMGB1 and RAGE enhanced the proliferation capability of the autophagy-deficient hepatocytes and tumors. However, RAGE expression was only found on ductual cells and Kupffer’s cells but not on hepatoctyes, suggesting that HMGB1 might promote hepatic tumor growth through a paracrine mode, which altered the tumor microenvironment. Finally, RNAseq analysis of the tumors indicated that HMGB1 induced a much broad changes in tumors. In particular, genes related to mitochondrial structures or functions were enriched among those differentially expressed in tumors in the presence or absence of HMGB1, revealing a potentially important role of mitochondria in sustaining the growth of autophagy-deficient liver tumors via HMGB1 stimulation.

## Introduction

Autophagy is an important mechanism regulating tumorigenesis. Its dysfunction due to external stress or genetic inactivation may lead to tumorigenesis. Indeed, liver-specific deletion of *Atg5* or *Atg7* (*Atg5*^Δ*Hep*^ or *Atg7*^Δ*Hep*^) causes hepatic tumorigenesis^1–4^. Similarly, reduced autophagic activity from constant activation of mammalian target of rapamycin complex 1 (mTORC1) also promotes hepatic neoplastic transformation^5,6^. These studies suggest that hepatocytes require the tumor-suppressive function of autophagy for maintaining its homeostasis.

Excessive reactive oxygen species (ROS) generated due to autophagy-deficiency is implicated in tumor development^7,8^. Consequently, pharmacological inhibition of ROS formation by the antioxidant N-acetylcysteine results in a strong suppression of tumor development in *Atg5*-deficient liver^8^. Moreover, there is a persistent activation of an anti-oxidative stress-related transcription factor NRF2 (nuclear factor, erythroid 2 like 2) to limit the oxidative injury^9^. Paradoxically, codeletion of *Nrf2* gene also prevents tumorigenesis in the autophagy-deficient liver^1,3^.

In additon, autophagy can regulates hepatic tumorigenesis by modulating the release of a damage-associated molecular pattern (DAMP) molecule, HMGB1. We have shown that defective autophagy leads to NRF2-mediated activation of Caspase-1/11, which in turn causes HMGB1 release^2^. It is known that extracellular HMGB1 acts as an immune mediator in sterile inflammation. However, codeletion of *Hmgb1* in the autophagy-deficient liver results in delayed tumor development via an unknown mechanism independent of its usual role in inflammation and fibrosis^2^.

In the present study, we have characterized the cellular and molecular context of the hepatic tumors driven by autophagy deficiency. We showed that HMGB1 and its dominant receptor RAGE positively affect the proliferation of tumor cells, likely via a paracrine mode. RNA sequencing analysis suggested that the effect of HMGB1 can affect the expressional level of multiple genes, particularly those involved in mitochondrial structure and functions. Our data, therefore, identify a key role of HMGB1 in promoting autophagy-deficient tumor growth via novel mechanisms. HMGB1 could thus be a potential therapeutic target.

## Results

### Hepatic tumor cells in autophagy-deficient livers had features consistent with autophagy deficiency

Autophagy possesses both antitumorigenic and protumorigenic role, depending on whether it occurs before or after the onset of tumorigenesis. Autophagy-deficient livers develop tumors, confirming the surveillance role of autophagy in the liver. The tumor first appears at the 9-month of the age and the tumor size and the number gradually increase as the mice get older^2,3^. The tumors in the autophagy-deficient livers seem to be hepatic adenoma, which does not metastasize^3^. However, the molecular and cellular nature of these tumors had not been fully characterized.

**Fig. 1:**
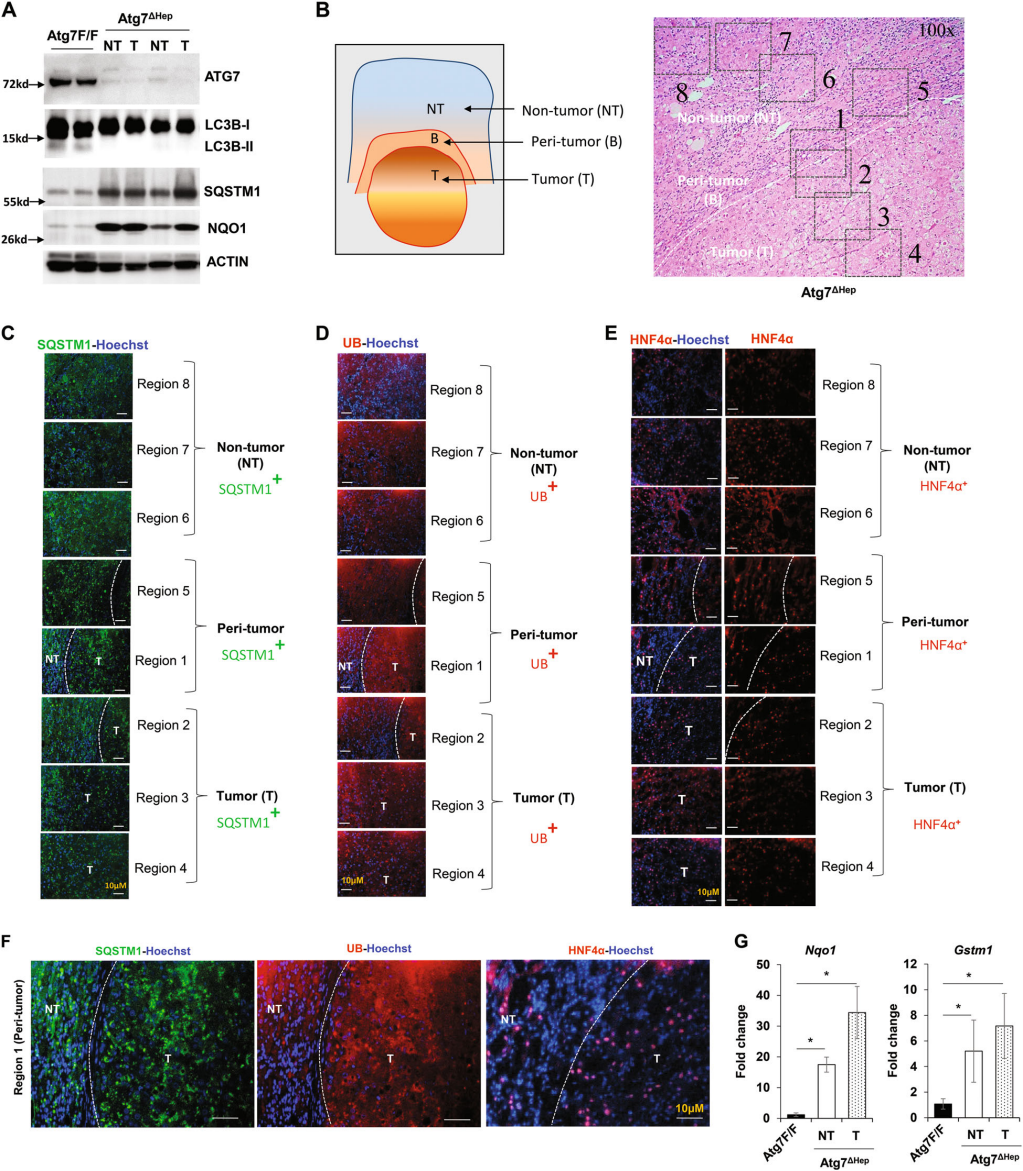
Hepatic tumor in autophagy-deficient livers are derived from autophagy-deficient hepatocytes. **a** Immunoblot analysis of autophagy function-related proteins (ATG7, SQSTM1, LC3B-I/II) and NRF2 pathway-related proteins (NQO1) in whole livers isolated from 15-month-old *Atg7F/F*, and *Atg7*^Δ*Hep*^ mice. **b** Schematic representation of the non-tumor, peri-tumor, and tumor region of the liver sections. Region 1 and Region 5: peri-tumor region, Region 2-Region 4: tumor region, and Region 6- Region 8: non-tumor region. **c**–**e** Livers from 12-month-old mice of *Atg7*^Δ*Hep*^ genotype were sectioned and immunostained with anti-SQSTM1(C), Anti-Ubiquitin (UB) (**d**), or anti-HNF4α (**e**). Dotted lines indicate the tumor border. **f** Magnified image of the region 1(peri- and intra-tumor region) of (**c**–**e**). **g** The hepatic mRNA expression level of NRF2 target genes, *Nqo1 and Gstm1*, in the livers of 15-month-old *Atg7F/F*, and in the non-tumor and tumor samples from the liver of age-matched *Atg7*^ΔHep^ mice. NT, non-tumor, T, tumor. Data are reported as mean ± SE, *P < 0.05; *n* = 3 mice per group.

Hepatic deletion of *Atg7* caused defective formation of LC3-II, an autophagy-specific marker, in tumor and non-tumor liver tissue, when compared with age-matched *Atg7-floxed* (*Atg7 F/F*) liver (Fig. 1a), indicating that the tumors were also deficient in autophagy and that they would have arisen from the autophagy-deficient hepatocytes. We confirmed this notion by examining the expression of SQSTM1 and ubiquitin (UB) in the liver. Immunohistological and immunofluorescence analysis was performed by taking images of eight different regions covering the non-tumor, peri-tumor, and the tumor regions as shown in Fig. 1b. A clear accumulation of SQSTM1 and UB in the tumor region of the autophagy-deficient liver was observed, which was at the level similar to that in the non-tumor tissues (Fig. 1c, d), suggesting that the tumor tissues were defective in autophagy and had defective protein quality control. In addition, the tumor tissues were positive for the hepatocyte-specific marker, HNF4α, which was colocalized in the same cells that had elevated SQSTM1 and UB staining (Fig. 1f).

We next analyzed whether the accumulation of SQSTM1 in tumor tissue could activate the anti-oxidative response-related NRF2 transcription factor as in non-tumor tissues^1,9^. We found that the protein and the mRNA level of *Nqo1* and *Gstm1* (NRF2 target genes) were drastically elevated in the tumor tissues of the *Atg7*^Δ*Hep*^ mice (Fig. 1a, g). These observations indicated that hepatic tumors in autophagy-deficient livers arise from the autophagy-deficient hepatocytes with upregulated NRF2 and SQSTM1 levels.

### Hepatic progenitor cells were localized exclusively in the non-tumor region but not inside the tumor

Hepatic progenitor cells (HPCs), also known as oval cells or ductular cells, expand during chronic liver injury in patients and in rodents^10,11^. The expansion of HPCs is significant in the autophagy-deficient livers^2^. HPCs has been noted to possess the capacity to become tumorigenic in vivo^12^. We thus explored the relationship of these cells to the tumor in autophagy-deficient livers by examining their spatial interactions.

**Fig. 2:**
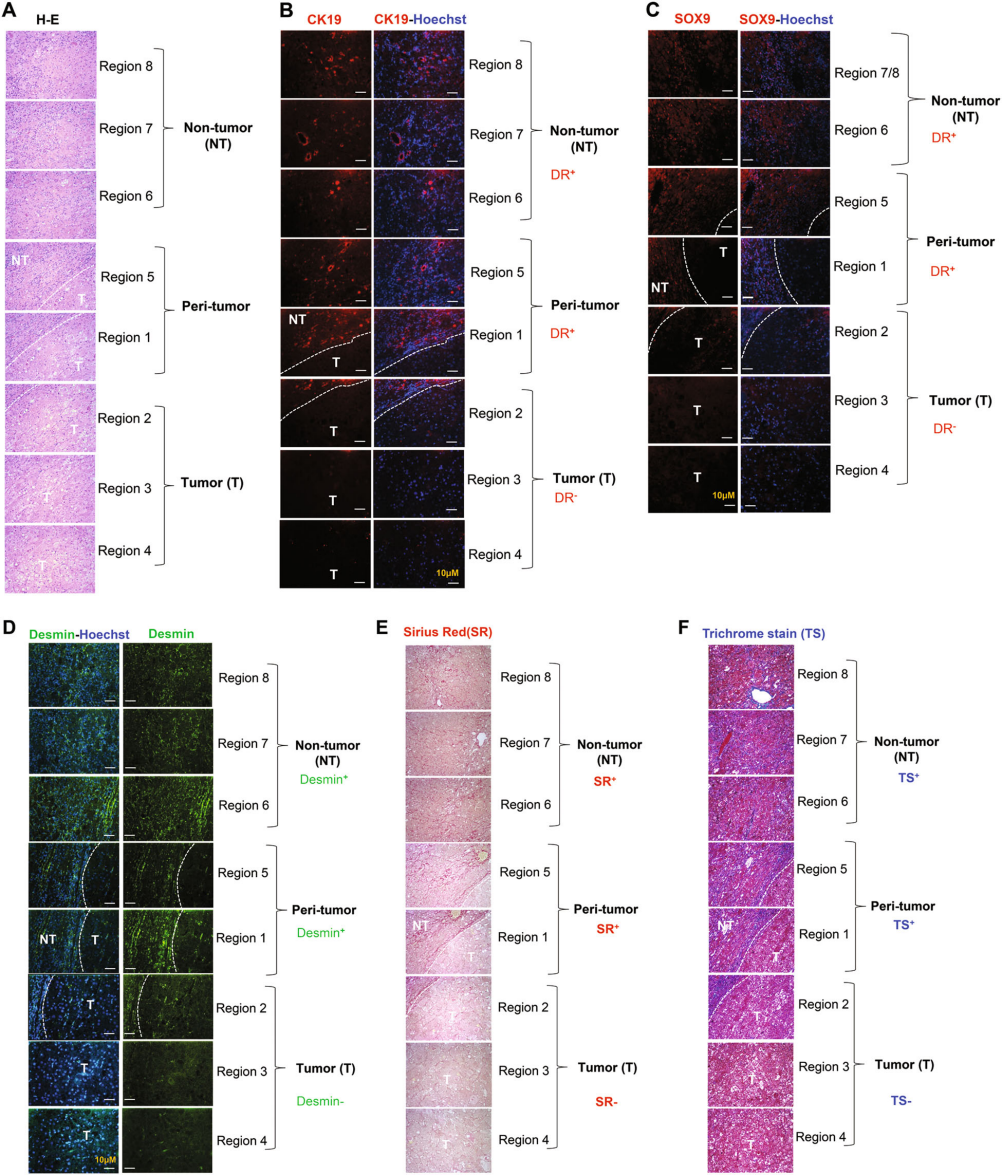
Hepatic progenitor cells and fibrosis are localized exclusively in peri-tumor and non-tumor regions but are absent inside the tumor. Liver sections from 12-month-old mice of the *Atg7*^Δ*Hep*^ genotype were subjected to H-E staining (**a**) (original magnification, ×200) and immunostaining for CK19 (**b**), SOX9 (**c**), Desmin immunostaining (**d**), Sirius Red stain (**e**), or Trichrome stain (**f**) (original magnification, ×200). Dotted lines indicate the tumor border. NT, non-tumor, T, tumor.

H-E staining showed that the distribution of HPCs was mostly around the tumor-adjacent region (Fig. 2a). In the area of tumor tissues, the normal tissue architecture, such as bile duct, and portal tract formation, was completely lost. Moreover, the tumor region was composed of irregular hepatic plates with tumor cells showing large nuclear-cytoplasmic ratio and occasionally nuclear atypia (Fig. 2a). Immunostaining for CK19 and Sox9, common markers for expanded HPCs, was negative in the tumor (Fig. 2b, c). Instead, most of the CK19- or Sox9 positive cells appear to form a compact sheet surrounding the tumor (Fig. 2b, c). Some of the HPCs were positive for SQSTM1 aggregates (Supplementary Fig. S1A–B). The possibility that some of these SQSTM1 positive HPCs may be derived from the autophagy-deficient hepatocytes cannot be excluded as such transdifferentiation had been reported previously^10,13^.

Interestingly, HPC and liver cancer stem cells (CSCs) also share several cellular markers, such as EpCAM, CD133 and CD24^14,15^. HPCs in the context of chronic liver injury have also been considered as a possible origin of liver CSCs. We thus analyzed the expression of these CSC markers in the non-tumor and tumor tissues of the autophagy-deficient liver. Real-time PCR analysis showed that the expression of *Cd133*, *Cd200*, *Cd34*, *Cd44*, *Ly6a/Sca1* and *Ly6d*, but not *Cd24a* and *Cd90*, were significantly upregulated in *Atg7*^Δ*Hep*^ livers compared with control *Atg7 F/F* livers (Supplementary Fig. S2A). The elevation of these CSC markers in the tumor tissues also suggested that tumors had a precursor/stem-cell phenotype. Interestingly, most of the stemness-related transcription factors such as *Oct4*, *Nanog*, *Klf4* and *Sox2* were significantly downregulated in *Atg7*^Δ*Hep*^ livers (Supplementary Fig. S2B). The lack of expression of Nanog has been linked to the adenoma nature of the tumor^16^. These changes were not more significant in the tumor tissue than in the non-tumor tissues, and thus may not be the mechanisms discriminating the two types of tissues.

HPCs have been reported to express multiple angiogenic paracrine factors such as vascular endothelial growth factor (VEGF), platelets-derived growth factor (PDGF), and angiopoietin (ANGPT) in pediatric hepatoblastoma^17^. These HPCs could interact with pro-tumorigenic cells heterotypically via mitogenic factors. Indeed, real-time PCR analysis indicated that expression of *Angpt2* and *Pdgfb*, but not *Vegfa* and *Angpt1*, were significantly upregulated in *Atg7*^Δ*Hep*^ tumor and non-tumor livers, compared with wild-type *Atg7 F/F* mice (Supplementary Fig. S3), suggesting the differential involvement of angiogenic factors in the peritumoral niche of *Atg7*^Δ*Hep*^ mice. Taken together, the distinct separation of the HPCs and tumor cells in the *Atg7*^Δ*Hep*^ livers suggests that HPCs may not evolve into the tumor cells but could contribute to a tumor microenvironment that affects tumorigenesis.

### Fibrosis was present in the peri-tumor region and encapsulated the tumor

Development of hepatic tumors are strongly associated with fibrosis, with 80–90% of HCCs developing in the fibrotic or cirrhotic livers^18^. On a cellular level, fibrogenesis is most significantly mediated by the activation of hepatic stellate cells (HSCs). Liver fibrosis occurs early in the autophagy-deficient liver^2^. In the older tumor-bearing *Atg7*^Δ*Hep*^ livers the number of desmin-expressing HSCs was still at an elevated level (Fig. 2d). Unlike the distribution of macrophages, desmin-positive HSCs were only present in the non-tumor and peri-tumor regions of the liver, but not inside the tumor (Fig. 2d). Consistently, increased fibrillar collagen deposition was only detected in the non-tumor and peri-tumor region (Fig. 2e, f). Taken together, the peri-tumoral desmin-positive HSCs may be responsible for the production of the fibers that encapsulated and demarcated the tumor tissue. It is possible that fibrosis in the autophagy-deficient liver may play an inhibitory role against tumor infiltration into normal tissues, thus contributing to the more benign presentation of the tumorigenesis in this setting.

### Macrophages but not other immune cells were found inside the tumor

**Fig. 3:**
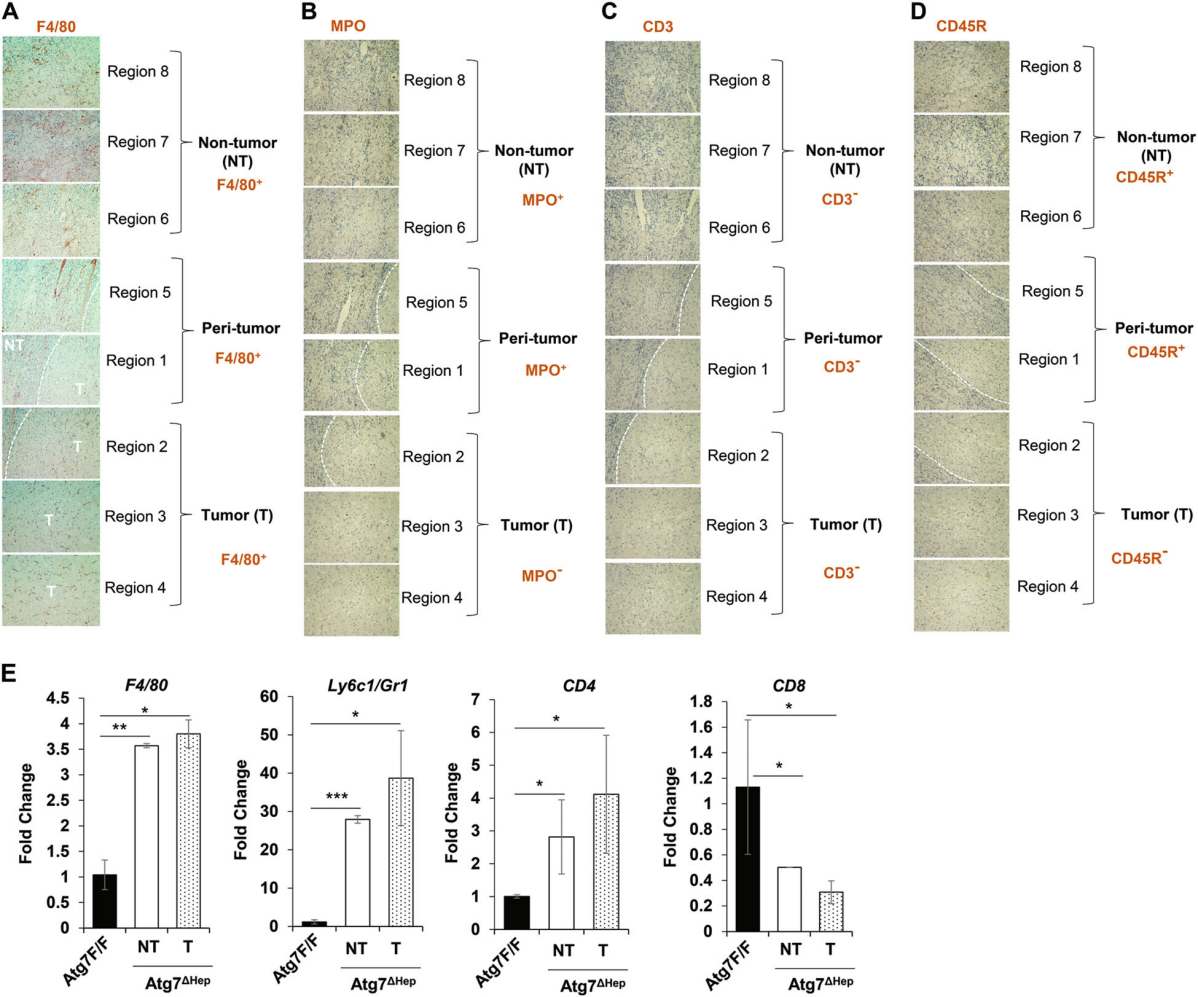
Macrophages but not other immune cells are found within the tumor. Liver sections from 12-month-old mice of *Atg7*^Δ*Hep*^ genotype were subjected to immunohistochemistry staining for F4/80 (**a**), Myeloperoxidase (MPO) (**b**), CD3 (**c**) and, CD45R (**d**) (original magnification, ×100). Dotted lines indicate the tumor border. (**e**) The hepatic mRNA expression level of immune cell-associated genes in 15-month-old *Atg7F/F* and *Atg7*^Δ*Hep*^ liver tissues. NT, non-tumor, T, tumor. Data are reported as mean ± SE, *P < 0.05, **P < 0.01, ***P < 0.001, n.s.: no significance; *n* = 3 mice per group.

Hepatocellular neoplasia often occurs in the setting of chronic inflammation, which is present in autophagy-deficient livers^2,19^. Among many different types of inflammatory cells, the tumor-associated macrophage (TAM) are thought to contribute to the initiation and promotion of tumors via cytokine factors. We thus examined the distribution of hepatic F4/80-positive cells, which showed their presence in both tumoral and non-tumoral regions (Fig. 3a). In contrast, most of the myeloperoxidase (MPO)-positive neutrophils, CD3-positive T cells, and CD45R-positive B cells were absent from the tumoral region but present exclusively in the non-tumor region (Fig. 3b–d). qRT-PCR analysis also found a strong upregulation of F4/80 and Ly6c expression in 12-month-old *Atg7*^Δ*Hep*^ livers as compared with age-matched *Atg7F/F* livers, and there was a further elevation in tumor tissues (Fig. 3f). The CD4 mRNA level was modestly elevated but the CD8 mRNA level was significantly suppressed in the tumor-bearing *Atg7*^Δ*Hep*^ liver (Fig. 3e).

Macrophages can play important roles in regulating hepatocytes proliferation and survival by secreting inflammatory cytokines, resulting in enhanced tumor growth^20^. In contrast to the presence of infiltrating F4/80-positive macrophages and the elevated expression of F4/80 and Ly6c, the mRNA expression of *TNFα*, *IL-6*, *Il-1β*, and *IL-17* were paradoxically downregulated in the tumor-bearing 12-month-old *Atg7*^Δ*Hep*^ livers (Supplementary Fig. S4). These data suggest that there is ongoing non-resolving inflammation in tumor and non-tumor tissue of autophagy-deficient mice but their contribution to tumor growth has yet to be fully determined. Alternatively other types of cytokines could be involved.

### Autophagy deficient hepatic tumor cells released HMGB1

Autophagy-deficient hepatocytes continuously release HMGB1, which impacts the expansion of HPCs^2^. HMGB1 might recruit inflammatory cells or fibrotic cells to the tumor region, promoting a permissive microenvironment^21,22^. We thus sought to determine whether the autophagy-deficient tumor tissues also release HMGB1, which might result in a positive feedback enhancement.

**Fig. 4:**
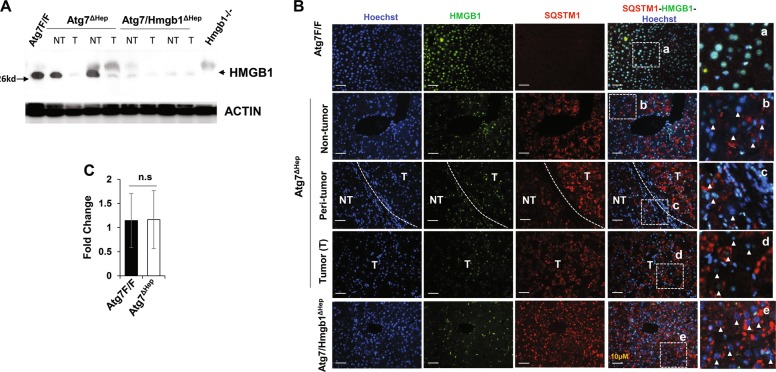
Hepatic HMGB1 is absent in the tumor of autophagy-deficient livers. **a** Livers of 15-month-old mice of different genotypes were examined for HMGB1 by immunoblotting assay. **b** Liver sections from 15-month-old mice of different genotypes were immunostained with anti-HMGB1 and anti-SQSTM1. White dotted lines indicate the tumor border. White arrowhead indicates the hepatocytes without nuclear HMGB1. **c** The hepatic mRNA expression level of *Hmgb1* in 15-month old-*Atg7F/F* and *Atg7*^Δ*Hep*^ mice, determined by real-time PCR. NT, non-tumor, T, tumor. Data are reported as mean ± SE, n.s., no significance; *n* = 3 mice per group.

We found that less HMGB1 proteins were present in tumor and non-tumor tissue of the *Atg7*^Δ*Hep*^ liver, as compared with the *Atg7 F/F* liver (Fig. 4a). Co-immunofluorescence staining also showed that tumor cells with accumulated SQSTM1 were also devoid of both nuclear and cytosolic HMGB1 (Fig. 4b). The mRNA level of HMGB1 was comparable between the liver tissues of floxed and *Atg7*^Δ*Hep*^ mice (Fig. 4c). Thus, the results indicated that autophagy-deficient tumor cells had released HMGB1.

### HMGB1 promoted hepatic proliferation

**Fig. 5:**
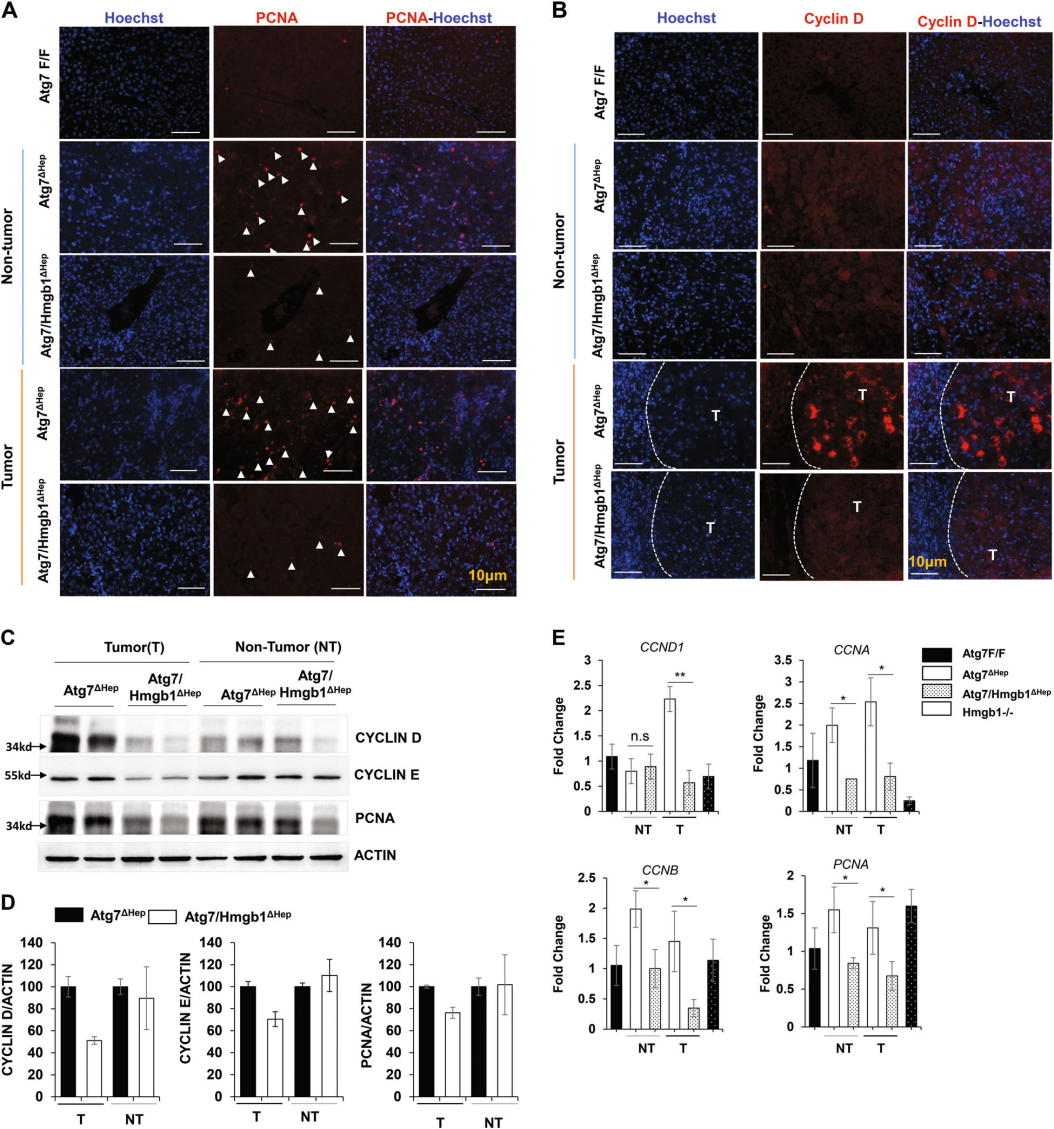
Loss of HMGB1 in hepatocytes correlates with reduced proliferation in the tumor. **a**, **b** Liver sections from 15-month-old mice of different genotypes were immunostained with anti-PCNA (**a**), or anti-Cyclin D (**b**). White arrow indicated proliferating hepatocytes. White dotted lines indicate the tumor border. **c** Immunoblot analysis of PCNA, cyclin D1, and cyclin E proteins in the tumor or non-tumor sample of 15-month-old *Atg7*^Δ*Hep*^ and, *Atg7/Hmgb1*^Δ*Hep*^ mice. **d** Densitometry qualification of the indicated proteins. **e** The hepatic mRNA level of indicated genes were determined in the indicated tissues of 15-month-old mice of different genotypes, determined by real-time PCR. NT, non-tumor, T, tumor. Data are reported as mean ± SE, *P < 0.05, **P < 0.01, n.s., no significance; *n* = 3 mice per group.

HMGB1 has a mitogenic effect in human HCC cell lines^23^. HMGB1 released by autophagy-deficient hepatocytes affected the growth of tumorigenic hepatocytes^2^. Consistently, we now found that *Atg7*^*ΔHep*^ livers had a remarkably increased number of hepatocytes positive for proliferation of cell nuclear antigen (PCNA) or Ki67, which seemed to be present in both non-tumor and tumor regions without much differences in the level (Fig. 5a) (Supplementary Fig. S5A, B). We then compared the cellular proliferation in 15-month-old *Atg7*^Δ*Hep*^ and *Atg7/Hmgb1*^Δ*Hep*^ mice to determine the role of HMGB1. Both genotypes developed a notable but different number of tumors at this age^2^. Immunostaining analysis for PCNA showed a lower number of proliferating hepatocytes in the tumor and non-tumor regions of *Atg7/Hmgb1*^Δ*Hep*^ livers than those in the *Atg7*^Δ*Hep*^ livers (Fig. 5a), suggesting that HMGB1 contributed to an overall enhanced proliferation status in autophagy-deficient livers.

Further supporting this notion, we found that the expression of Cyclin D1 was more significantly upregulated in the tumor of *Atg7*^Δ*Hep*^ liver than in the tumors of the *Atg7/Hmgb1*^Δ*Hep*^ livers (Fig. 5B, Supplementary Fig. S6). Immunoblot analysis of Cyclin E also showed a higher level in tumor and non-tumor regions of the *Atg7*^Δ*Hep*^ livers than that in the *Atg7/Hmgb1*^Δ*Hep*^ livers (Fig. 5c, d). Real-time PCR analysis demonstrated that hepatic expression of *CCND1*, *CCNA1*, and *CCNB1* were significantly upregulated in *Atg7*^Δ*Hep*^ mice, compared with *Atg7 F/F* mice (Fig. 5e). The expression of *CCND1* and *CCNA1* was even more prominently elevated in the tumor region than in the non-tumor tissues in *Atg7*^Δ*Hep*^ mice (Fig. 5e). Such induction was not observed in tumor tissues from *Atg7/Hmgb1*^Δ*Hep*^ mice (Fig. 5e), suggesting that *Hmgb1* deletion retarded cell cycle progression via the downregulation of the expression of cyclins in the autophagy-deficient livers. These results indicated that hepatic tumors of *Atg7/Hmgb1*^Δ*Hep*^ were less proliferative than the tumors in *Atg7*^Δ*Hep*^ mice. Thus HMGB1 had an impact on cell proliferation in the autophagy-deficient liver.

We then examined various cell growth relates signaling pathways such as the PI3K/AKT pathway, the JNK pathway, the mTORC1 pathway, the MAPK/ERK pathway, and the JAK/STAT3 pathway, that regulates various cellular responses in HCC proliferation and survival^24–29^. Intriguingly, immunoblot analysis showed that phospho-AKT and phospho-JNK was detected at higher levels in *Atg7/Hmgb1*^Δ*Hep*^ livers compared with *Atg7*^Δ*Hep*^ livers regardless the sample type (Supplementary Fig. S7A, B). However, we did not detect significant differences in the activation of other pathways related proteins between *Atg7*^Δ*Hep*^ mice and *Atg7/Hmgb1*^Δ*Hep*^ mice (Supplementary Fig. S7C–E). Taken together while the reason for the paradoxical elevation of AKT and JNK phosphorylation in *Atg7/Hmgb1*^Δ*Hep*^ livers is not clear these events do not seem to be tumor specific and may not be related to the reduced proliferation status of tumors from in these livers. Alternativley, it is notable that hepatocytes could offer a very different cellular context in which the conventional oncogenes or tumor suppressor genes can act in opposite ways^30,31^.

### RAGE deletion impairs proliferation and retards liver tumor development

**Fig. 6:**
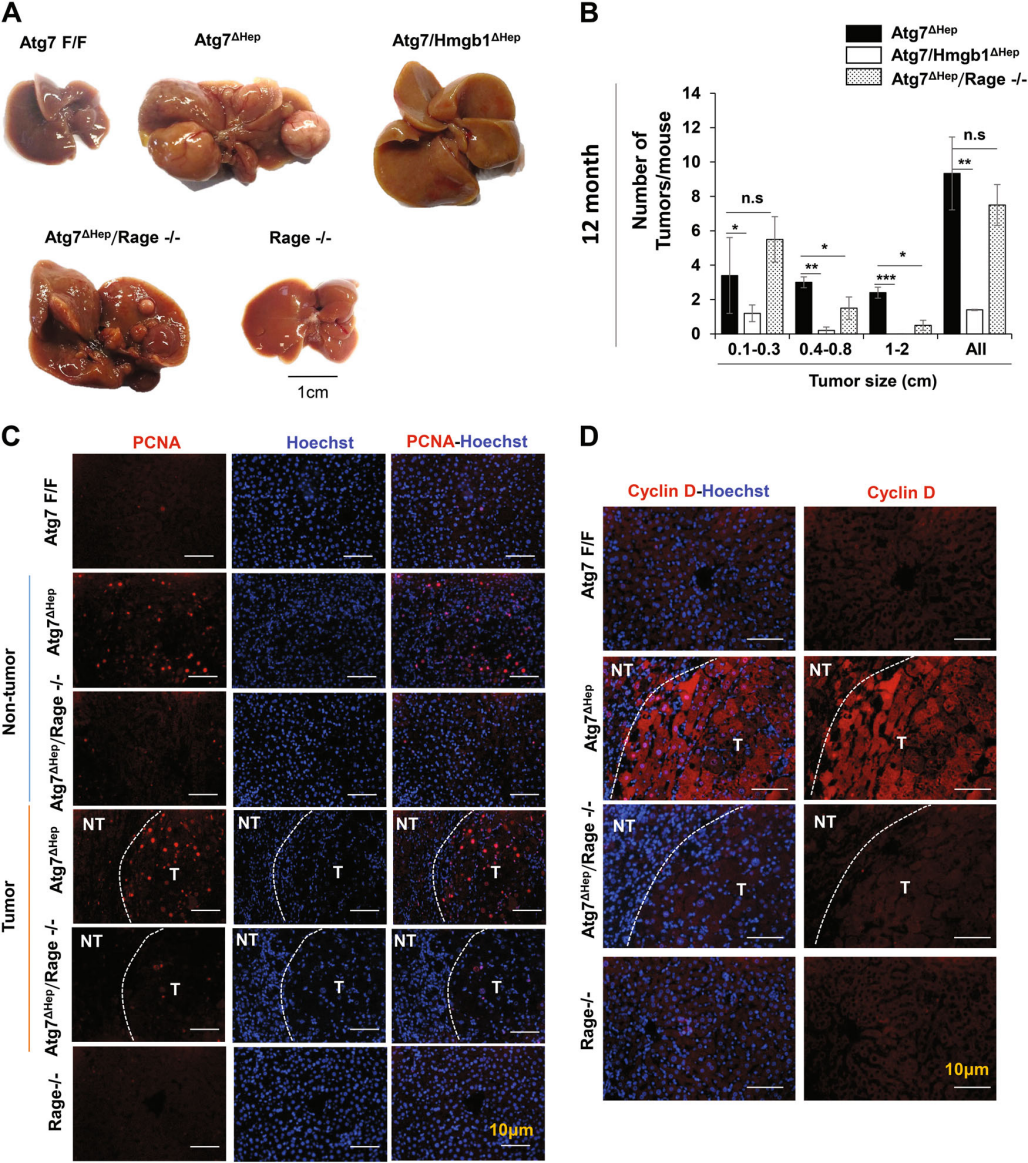
Genetic loss of *Rage* inhibits tumorigenesis in autophagy-deficient livers. **a** Gross images of representative livers of 12-month-old *Atg7*^Δ*Hep*^, *Atg7/Hmgb1*^Δ*Hep*^, *Atg7*^Δ*Hep*^*Rage-/-*, and *Rage-/-* mice. **b** Average number and size distribution of the tumors observed in the livers of 12-month-old mice of different genotypes. **c–d** Liver sections from 12-month-old mice of different genotypes were immunostained with anti-PCNA (**c**), or anti-Cyclin D (**d**). White dotted lines indicate the tumor border. NT. non-tumor, T, tumor. Data are reported as mean ± SE, *P < 0.05, **P < 0.01, ***P < 0.001, n.s., no significance; *n* = 3 mice per group. Size information of the tumor from *Atg7/Hmgb1*^Δ*Hep*^ *livers* is derived from what we has previously reported^2^.

Extracellular HMGB1 can binds to RAGE or TLR4^32^. In our previous study, *Atg7*^*ΔHep*^ mice develop hepatic tumors at 9-month old, which was inhbited by the deletion of either *Hmgb1* or *Rage*^2^. While *Atg7/Hmgb1*^Δ*Hep*^ at the age of 12-month old were still largely devoid of tumors in the liver^2^, we now found that 12-month-old *Atg7*^Δ*Hep*^*/Rage-/-* mice developed a significant presence of tumors (Fig. 6a), which, however, were significantly smaller in size compared with those in the *Atg7*^Δ*Hep*^ mice (Fig. 6b). Notably, the number of PCNA-positive cells and the expression of cyclin D1 were also remarkably decreased in *Atg7*^Δ*Hep*^*/Rage-/-* livers compared with that in the *Atg7*^Δ*Hep*^ livers(Fig. 6c–d). These data suggest that the loss of *Rage* in autophagy-deficient livers reduced tumor cell proliferation and tumor expansion in the liver. HMGB1 interaction with the RAGE receptor can thus mediate a significant level of cell proliferation and tumor development in the autophagy-deficient liver.

**Fig. 7:**
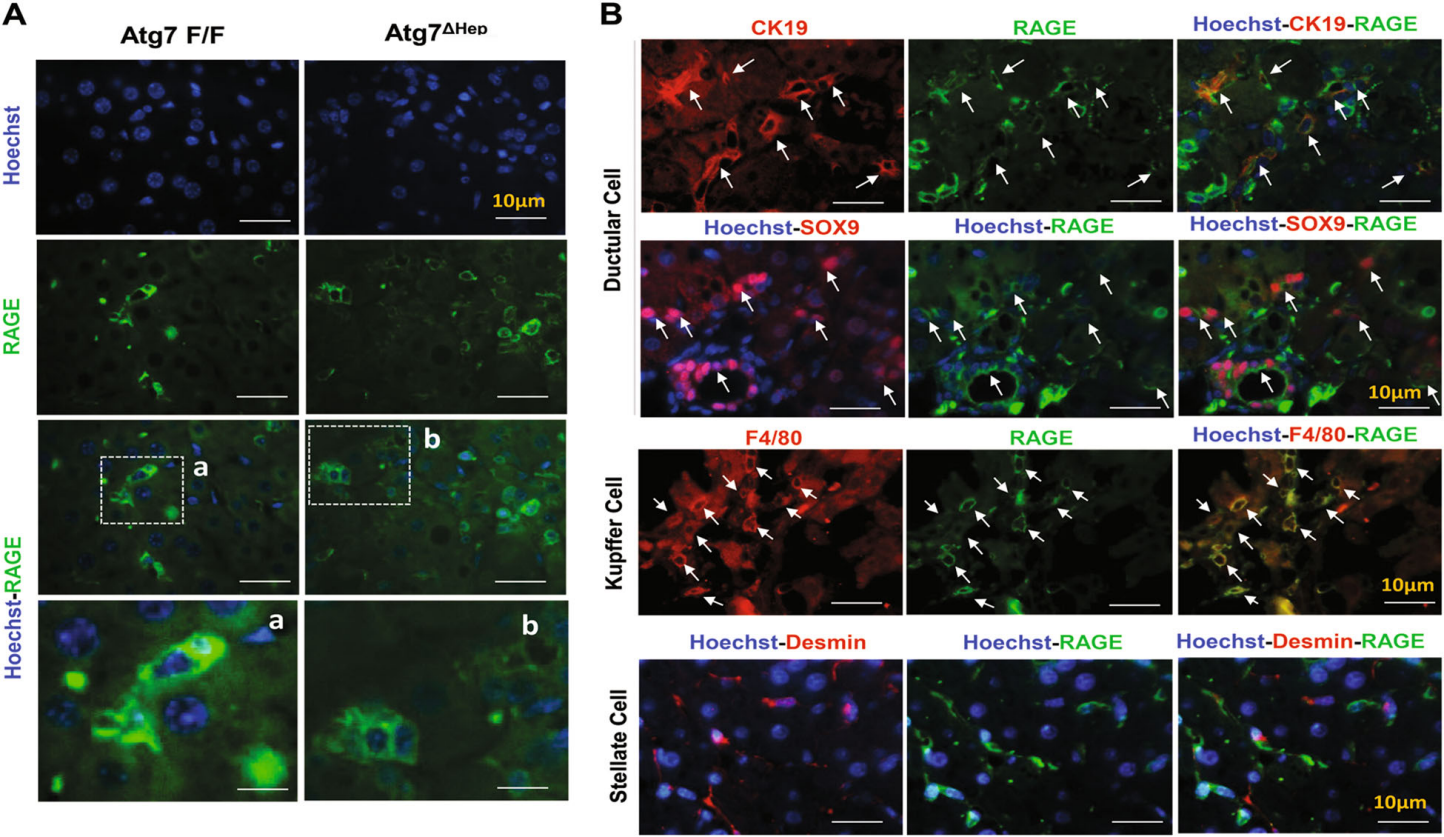
RAGE is expressed by ductular cells and Kupffer’s cells but not by hepatocytes or stellate cells. **a** Immunofluorescence staining for RAGE antigen in the livers of 9-week-old mice of *Atg7F/F* and *Atg7*^Δ*Hep*^genotype. Framed areas are enlarged and shown in separate panels (**a**, **b**). **b** Liver sections from 9-week-old *Atg7*^Δ*Hep*^mice were coimmunostained with anti-RAGE, together with anti-CK19 or SOX9 or F4/80 or Desmin. White arrows indicate cells with colocalized signals.

To determine whether HMGB1 released by the autophagy-deficient hepatocytes could act as an autocrine or paracrine fashion to promote cellular proliferation, we examined RAGE expression by immunofluorescence staining in frozen tissue from *Atg7 F/F* and *Atg7*^Δ*Hep*^ livers. We found that RAGE was almost exclusively expressed on the surface of cells other than hepatocytes based on cell morphology (Fig. 7a). Double immunofluorescence showed that colocalization of RAGE was evident in CK19 or SOX9-positive ductular cells and F4/80-positive Kupffer cells, but not on the Desmin-positive stellate cells in *Atg7*^Δ*Hep*^ liver (Fig. 7b).

These findings indicate that RAGE was expressed on ductular cells and Kupffer cells but not on hepatocytes nor stellate cells. Futhermore, these observations suggest that unlike the possible direct effect of HMGB1 on the expansion of CK19-positive or SOX9-positive ductual cells^2^, the tumor-promoting effect of HMGB1 may not be mediated by a direct effect on the autophagy-deficient hepatocytes, but possibly by an indirect effect through other RAGE-expressing cells, such as the Kupffer’s cells, which could then alter the microenvironment to facilitate tumor development.

### RNA sequencing revealed key molecular differences between tumors from *Atg7*^Δ*Hep*^ mice and from *Atg7/Hmgb1*^Δ*Hep*^ mice

Since the effect of HMGB1 in promoting tumor development may be at least in part mediated by an altered microenvironment, there could be multiple alterations in tumor behaviors affected by this process. We sought to investigate the transcriptomic profile of the tumor to better understand the impact of HMGB1 on tumor development in autophagy-deficient livers. We chose to perform RNA sequencing on tumor tissues obtained from *Atg7*^Δ*Hep*^ and *Atg7/Hmgb1*^Δ*Hep*^ mice at the age of 15-months old, when the tumor number and size were comparable in these mice.

**Fig. 8:**
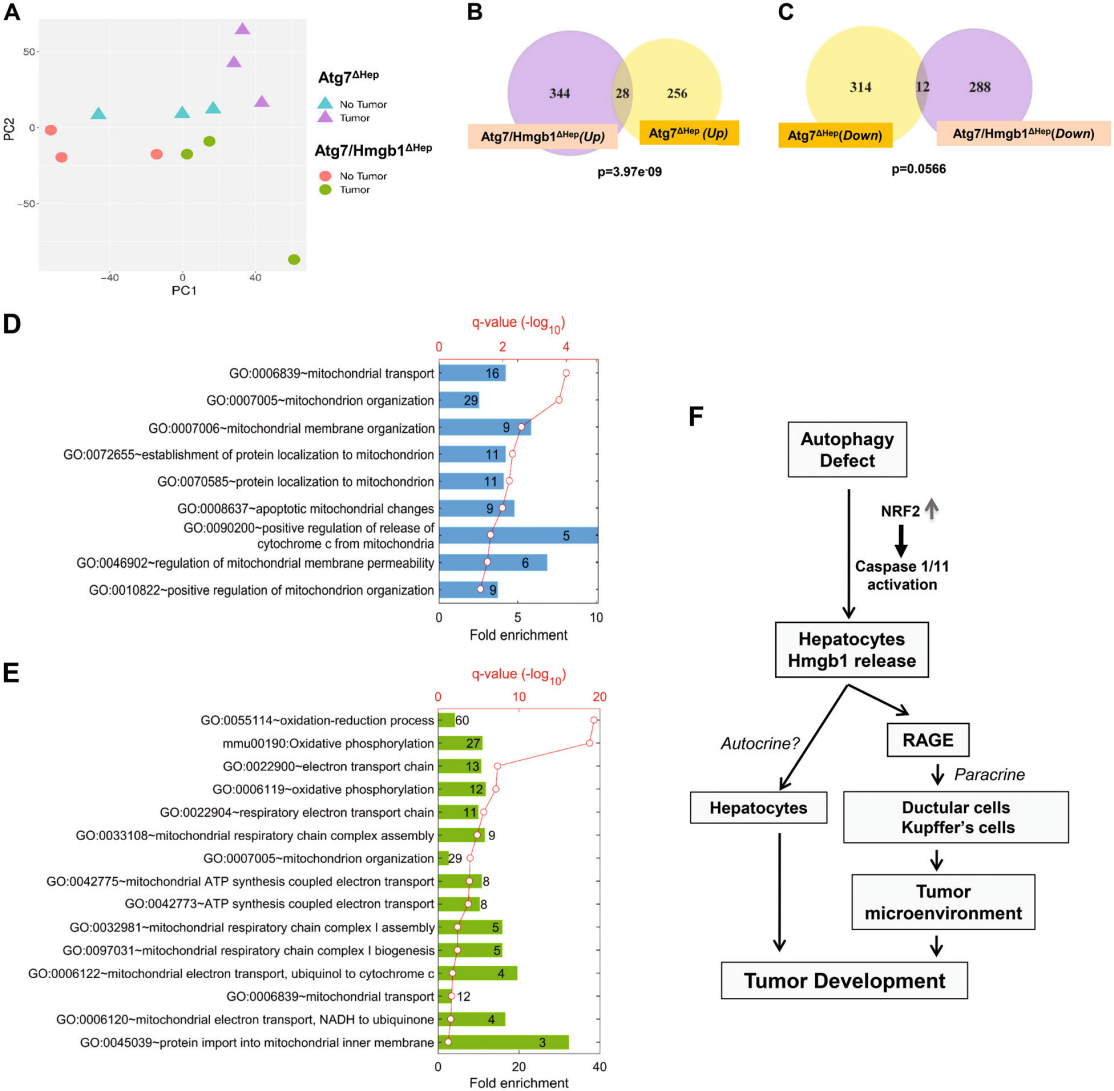
RNAseq analysis indicates transcriptomic differences in the hepatic tumors of Atg*7*^Δ*Hep*^mice and *Atg7/Hmgb1*^Δ*Hep*^ mice. **a** PCA of transcriptomic data based on 12 RNA-seq samples under the four indicated combinations of genotypes and tissue types. **b**–**c** Numbers of DEGs that are significantly upregulated (**b**) or downregulated (**c**) (*p* < 0.01) in the tumor samples of *Atg7/Hmgb1*^Δ*Hep*^ and/or *Atg7*^Δ*Hep*^mice. The *p*-values are indicated for the overlap between the two groups of upregulated or downregulated DEGs, respectively. **d** GO biological processes significantly over-represented in the non-overlapped 256 DEGs uniquely elevated in the tumor samples of the *Atg7*^Δ*Hep*^ mice. **e** GO biological processes and KEGG pathways significantly enriched in the non-overlapped 288 DEGs uniquely repressed in the tumor samples of the *Atg7/Hmgb1*^Δ*Hep*^ mice. For (**d**) and (**e**), the heights of bars indicate the fold enrichment compared with random selection, whereas the red dots represent the statistical significance, *p*-value after FDR-adjusted multiple test correction. The numbers in the bars represent the numbers of DEGs in the particular group which are associated with corresponding GO terms. **f** Schematic model for the role of HMGB1 in tumor development in the autophagy-deficient liver. HMGB1 is released from autophagy-deficient hepatocytes via the NRF2-inflammasome pathway. Deletion of RAGE, an HMGB1 receptor, mimicked the effect of HMGB1 deletion in delaying tumor development, suggesting that HMGB1 affects tumor development via its released form, but not its DNA-binding form. That HMGB1 may act on hepatocytes in an autocrine fashion could not be completely excluded although hepatocytes do not seem to express a detectable level of RAGE. Released HMGB1 could thus have paracrine effects on target cells that express RAGE and may affect tumor development by altering the microenvironment.

The principal component analysis (PCA) on the RNAseq data indicated different transcriptomic profiles in the tumor tissues of the two strains of mice when compared with the non-tumor tissues (Fig. 8a), The six non-tumor samples from the two strains of mice were close to each other. In addition, two out of the three tumor samples from *Atg7/Hmgb1*^Δ*Hep*^ livers were also close to the non-tumor samples whereas tumor samples from *Atg7*^Δ*Hep*^ mice were separated the farthest from the rest of the samples. PCA thus suggests that tumors from the two strains of mice were quite different with those from *Atg7/Hmgb1*^Δ*Hep*^ livers more similar to the non-tumor tissues in their transcriptomic profiles.

Differential expression analysis showed that 284 and 372 differentially expressed genes (DEGs) were upregulated in tumors of *Atg7*^Δ*Hep*^ and *Atg7/Hmgb1*^Δ*Hep*^ livers, respectively, whereas 326 and 300 genes were downregulated in tumors of these livers, respectively (Fig. 8b, c). A complete list of these DEGs can be found in Supplementary Tables S1–S5. We then focused on discovering unique molecular features in the tumors associated with the presence and absence of HMGB1. When comparing the DEGs between *Atg7*^Δ*Hep*^ and *Atg7/Hmgb1*^Δ*Hep*^ tumors, a small number of upregulated (28, Fig. 8b) or downregulated (12, Fig. 8c) DEGs were found in tumor tissues of both strains. The larger portions of DEGs were, however, unique in *Atg7*^Δ*Hep*^ and in *Atg7/Hmgb1*^Δ*Hep*^ tumors, supporting the notion that the tumors were different in the presence or absence of HMGB1.

To understand the molecular features of these differences, we determined the gene ontology (GO) terms and KEGG pathways that were significantly enriched in the unique DEG sets. We found that many biological processes, particularly those associated with mitochondrial structrures or functions were significantly over-represented by the uniquely upregulated DEGs in *Atg7*^Δ*Hep*^ tumors (Fig. 8d). Notably, DEGs that were uniquely downregulated in *Atg7/Hmgb1*^Δ*Hep*^ tumors were also enriched for those involved in the mitochondrial structures or functions, (Fig. 8f). Many genes related to mitochondrial oxidative phosphorylation (OXPHOS) or electron transport chain (ETC) process were significantly downregulated in the tumors of *Atg7/Hmgb1*^Δ*Hep*^ livers. Particulalry, the genes involved in the assembly or biogenesis of respiratory complex I (NADH dehydrogenase complex) and complex III (Ubiquinol to Cytochrome c electron transporter) were significantly downregulated. These observations suggested that a major component of the tumor-promoting effects of HMGB1 could be related to mitochondrial function and activity, which may impact the celluar bioenergetics and hence tumor growth in autophagy-deficient liver.

## Discussion

Autophagy is important for liver homeostasis and tumor surveillance. Deficiency of hepatic autophagy leads to tumor development in aged mice^1–4^. On the other hand, autophagy function is required for the aggressive growth of tumors. The mechanism that sustains the growth of autophagy-deficient tumors is not known. Our findings support the following conclusions: (1) The adenoma originates from the autophagy-deficient hepatocytes; (2) Hepatocyte-derived HMGB1 stimulates tumor cell proliferation; (3) HMGB1 mediates the proliferative signal at least in part via RAGE in a paracrine mode; and (4) Tumors developed in the presence or absence of HMGB1 have significantly different transcriptomic profiles and mitochondria function could be an important mechanistic linker to tumor promotion.

### 1. HMGB1 may act in a paracrine model to stimulate tumor growth

Histological analysis suggests that tumor cells were originated from the autophagy-deficient hepatocytes. The composition of the tumor appears to be different from the non-tumor liver tissue. In comparison to non-tumor tissues where different hepatic cells coexist, the tumor tissue consists of mainly the tumor cells (HNF4α positive), and some macrophages (Figs. 1–3) (Supplementary Table S6). Fibrotic cells and ductular cells seem to be responsible for the formation of a fibrous capsule that demarcates the tumor from the non-tumor tissue. How the autophagy-deficient hepatocytes form the adenomatous nodule, excluding the fibrotic cells and ductular cells but retaining some macrophages, is intriguing. But macrophages could belong to those known as TAM and may enter into the tumor tissue via tumor blood vessels^33^.

HMGB1 is known to promote tumor development^2,34,35^. HMGB1 has been shown to be important for expansion of ductular cells^2,34^, immune cells recruitment^22^ and activation of fibrotic cells^21^. All these cellular events could favor tumorigenesis. We now know that non-tumorigenic^2^ and tumorigenic (this study) autophagy-deficient hepatocytes both can release HMGB1. The proliferative effect of HMGB1 could be mediated via its receptor, RAGE (Fig. 6), although deletion of *Rage* was not as effective as deletion of *Hmgb1* to deter tumor development^2^ (Fig. 6a, b). It is possible that HMGB1 may affect the tumorigenesis process through other receptors, such as TLR4^32^. Future studies can assess the potential role of TLR4 in this process.

At the cellular level, RAGE is not expressed by hepatocytes and stellate cells at the detectable level by immunostaining. But it could be readily detected on the surface of Kupffer’s cells and HPCs^2,36^. Hence the effect of HMGB1 in cell proliferation could be mediated by a paracrine manner, although the autocrine mode could be not be completedly excluded (Fig. 8f). In the paracrine mode, HMGB1 released by hepatocytes could activate macrophages and/or ductular cells, which may then promote an intratumoral microenvironment favoring cell growth and proliferation. However, it seems that some of the well-defined proinflammatory cytokines such as TNFα, IL-1β, and IL-6 may not play the role as the expression of these cytokines were remarkably downregulated in tumor tissues (Supplementary Fig. S4). On the other hand, the RAGE-positive peri-tumoral ductular cells could possibly communicate with hepatocytes via cytokines such as angiogenic factors ANGPT2 and PDGFβ to promote angiogenesis tumor development. It is also possible that the protumorigenic factors from TAM and/or ductular cells could be mediated by other cytokines, chemokines, extracellular vesicles, microRNAs or other cellular factors^37^.

### 2. The impact of HMGB1 on tumor cells can be broad

Deletion of *Hmgb1* or *Rage* led to a signficant reduction in the proliferative capability of autophagy-deficient hepatocytes and tumors as demonstrated by the expression of PCNA, Ki67 and Cyclin D1. Thus the pro-proliferative effect by HMGB1 confers a generally stronger capability of proliferation to autophagy-deficient hepaotcytes, which would be benefical to the growth of tumors that are derived from these cells.

However, RNAseq analysis indicates that there are much more unique changes in the molecular composition of the tumors affected by HMGB1. The enrichment of certain gene expression related to mitochondrial structure and function in the presence of HMGB1 and lack of such enrichment in the absence of HMGB1 are quite significant. *Hmgb1* deletion appears to suppress the mitochondrial ETC in tumors of autophagy-deficient livers. Whether and how downregulation of genes of mitochondrial ETC may suppress cell proliferation in *Atg7/Hmgb1*^Δ*Hep*^ tumors is unclear. But it is well known that mitochondrial ETC enables many metabolic processes and is a major sources of ATP and building blocks for cellular activity. As a consequence of ETC dysfunction, cell proliferation could be impaired due to bioenergetics deficit. This notion is supported by the observation where pharmacological or genetic inhibition of ETC caused impaired cell proliferation of cells in vitro^38,39^. Interestingly, a recent study suggest that ETC enables aspartate biosynthesis, a key proteogenic amino acid that is also a precursor in purine and pyrimide synthesis and is required for tumor growth and survival^40,41^. Thus tumors in *Atg7/Hmgb1*^Δ*Hep*^ liver may have defective ETC that could impair cell proliferation by limiting an intracellular aspartate level besides having bioenergetics deficits. Many metabolic pathways including glycolysis, the TCA cycle, and β-oxidation produce the electron donors that fuel the ETC. Hence, impairment or downregulation of ETC could limit the regeneration of reducing equivalents, such as NAD+, which in turn suppresses glycolysis or the TCA cycle. Future studies should address these possibilities for the understanding of how HMGB1 sustains the growth of autoaphgy-deficient hepatic tumors.

In conclusion, our findings demonstrate that hepatic adenoma originates from the autophagy-deficient hepatocytes that release HMGB1. HMGB1, in turn, can stimulate hepatocyte proliferation and hepatic tumorigenesis via RAGE in the autophagy-deficient liver. The effect of HMGB1 on tumor cells are broad as revealed by transcriptomic analysis, which offers mechanistic clues for future studies.

## Materials and methods

### Animal experiments

*Atg7F/F*, *Atg7*^Δ*Hep*^, *Atg7/Hmgb1*^Δ*Hep*^, *Atg7*^Δ*Hep*^*Rage-/-*, *Hmgb1-/-*, and *Rage-/-* mice were used in this study. *Atg7F/F* was obtained from Dr. Komatsu Masaaki (Nigata University, Japan). These mice were backcrossed with C57BL/6J for another 10 generations as described previously^2,19^. Albumin-Cre mice were obtained from the Jackson Laboratory(Bar Harbor, ME). *Hmgb1 F/F* and *Rage* mice were as described^2^. *Atg7*^Δ*Hep*^mice were further crossed with *Hmgb1 F/F* or *Rage* to generate *Atg7/Hmgb1*^Δ*Hep*^or *Atg7*^Δ*Hep*^*/Rage-/-* mice as previously described^2^. Both male and female mice were used in the study. All animals received humane care, and all procedures were approved by the Institutional Animal Care and use Committee (IACUC) of the Indiana University.

### Tumor sample collection

The whole liver was carefully removed from the euthanized animals, washed, and placed in cold PBS. The number of tumor nodules on the liver surface was counted for all the liver lobes. Tumor nodules with >2 mm in diameter were carefully removed and examined as tumor tissue. Tissue without visible tumor nodules were sampled as non-tumor tissues. All tissues were collected in separate tubes and stored at −80 ^o^C for future studies. Liver tissues containing the tumor nodule and the surrounding non-tumor tissue were excised and fixed in 10% neutral formalin or buffered with 4% PFA overnight for paraffin-embedding or for OCT embedding. The tissue section was prepared from the frozen or paraffin blocks for general histology, immunostaining, and immunohistochemistry analysis.

### General histological and immunological analysis

General histology was examined on paraffin-embedded sections stained with hematoxylin and eosin (H-E). Liver fibrosis was determined by Sirius Red staining or Masson’s Trichome staining. For immunostaining, liver sections were subjected to heat-induced antigen retrieval using citrate buffer (pH 6.0) followed by permeabilization and blockage with 10% goat or donkey serum in PBS containing 0.5% triton-X for 1 h. Sections were incubated overnight at 4 ^o^C with primary antibody diluted in PBS. Primary antibodies used in this study are listed in Supplementary Table S8. Sections were then incubated with Alexa-488 or Cy3-conjugated secondary antibodies. Images were obtained using Nikon Eclipse TE 200 epi-immunofluorescence microscope. Hoechst 33342 was used for nucleus staining. Images were analyzed using NIS-element AR3.2 software.

Immunoblot analysis was performed as described previouosly^2,19^ using primary antibodies and respective secondary antibodies conjugated with horseradish peroxidase as listed in Supplementary Table S8. The respective protein bands were visualized using the immunobilion chemiluminescence system (Millipore, MA). The densitometry analysis of immunoblotting images was performed using Quantity One Software (Bio-rad). Densitometry values were normalized to the loading control (GAPDH) and then converted to units relative to the untreated control.

### Total RNA isolation, reverse transcription, and quantitative real-time PCR analysis

Total RNA was isolated from liver tissues using a GeneTET RNA Purification Kit (Thermo Fisher Scientific) according to the manufacturer’s protocol. cDNA was synthesized using an M-MLV Reverse Transcriptase Enzyme System (Life Technologies, Thermo Fisher Scientific) and OligodT primers. The resulting cDNA products were subjected to qPCR reaction using SYBR Green Master Mixes. qPCR was performed on a Quanta studio 3 PCR machine (Life Technologies–Applied Biosystems, Thermo Fisher Scientific). The threshold crossing value(Ct) was determined for each transcript and then normalized to that of the internal gene transcript(β-actin). Fold change values were then calculated using the 2^–ΔΔCt^ method. Genes-specific primers were designed using Integrated DNA Technologies (IDT) PrimerQuest software. Sequences of the forward and reverse primers are listed in Supplementary Table S7.

### RNA-sequencing and bioinformatics analysis

RNA was isolated as described above. RNAseq was performed by The Center for Medical Genomics facility at Indiana University. The integrity of RNA was determined using an Agilent Bioanalyzer 2100 (Agilent Technologies;Santa Clara, CA). Extracted RNA was processed for rRNA removal using the Epicenter rRNA depletion kit according to the manufacturer’s instructions. rRNA-depleted RNA was subsequently used to generate paired-end sequencing libraries using the Illumina RNA TruSeq Library Kit according to the manufacturer’s instruction. RNAseq was performed using Illumina HiSeq 4000 (Illumina, San Diego, CA). For bioinformatics analysis, we first used FastQC (http://www.bioinformatics.babraham.ac.uk/projects/fastqc) to examine RNA-seq quality. Then all high-quality sequences were mapped to the mouse genome (mm10, UCSC Genome Browser, https://genome.ucsc.edu/) with the STAR, an RNA-seq aligner^42^. The featureCounts was adopted to assign uniquely mapped reads to genes according to UCSC refGene (mm10)^43^. Those low-expressed genes were not further analyzed if their raw counts were less than 10 in more than three samples for each pairwise comparison. The gene expression was normalized cross all samples based on trimmed mean of M (TMM) values implemented in EdgeR^44^, followed by differential expression analysis given comparisons between non-tumor and tumor tissues, in either single knockout or double knockout mice. Genes with *p* values < 0.01 after multiple-test false discovery rate (FDR) correction were determined as DEGs for specific comparisons. The GO and KEGG pathways significantly enriched in DEGs were identified by DAVID functional annotation analysis tools^45^.

### Statistical analysis

Statistical analyses were performed with Sigma Plot. All experimental data were expressed as Mean ± SE. Student *t*-test was performed to compare values from two groups. To compare values obtained from three or more groups, one-way ANOVA analysis with the appropriate post-hoc analysis was used. Statistical significance was taken at the level of *P* < 0.05.

## Supplementary information

Supplementary Figure-S1

Supplementary Figure-S2

Supplementary Figure-S3

Supplementary Figure-S4

Supplementary Figure-S5

Supplementary Figure-S6

Supplementary Figure-S7

Supplementary Figure legends

Supplementary Table S1

Supplementary Table S2

Supplementary Table S3

Supplementary Table S4

Supplementary Table S5

Supplementary Table S6

Supplementary Table S7

Supplementary Table S8
