# Supplementary figures and images for: The HMGB1-RAGE axis modulates the growth of autophagy-deficient hepatic tumors
Source: Cell Death Dis. 2020 May 7;11(5):333. doi: 10.1038/s41419-020-2536-7 (PMC7206028; doi:10.1038/s41419-020-2536-7)

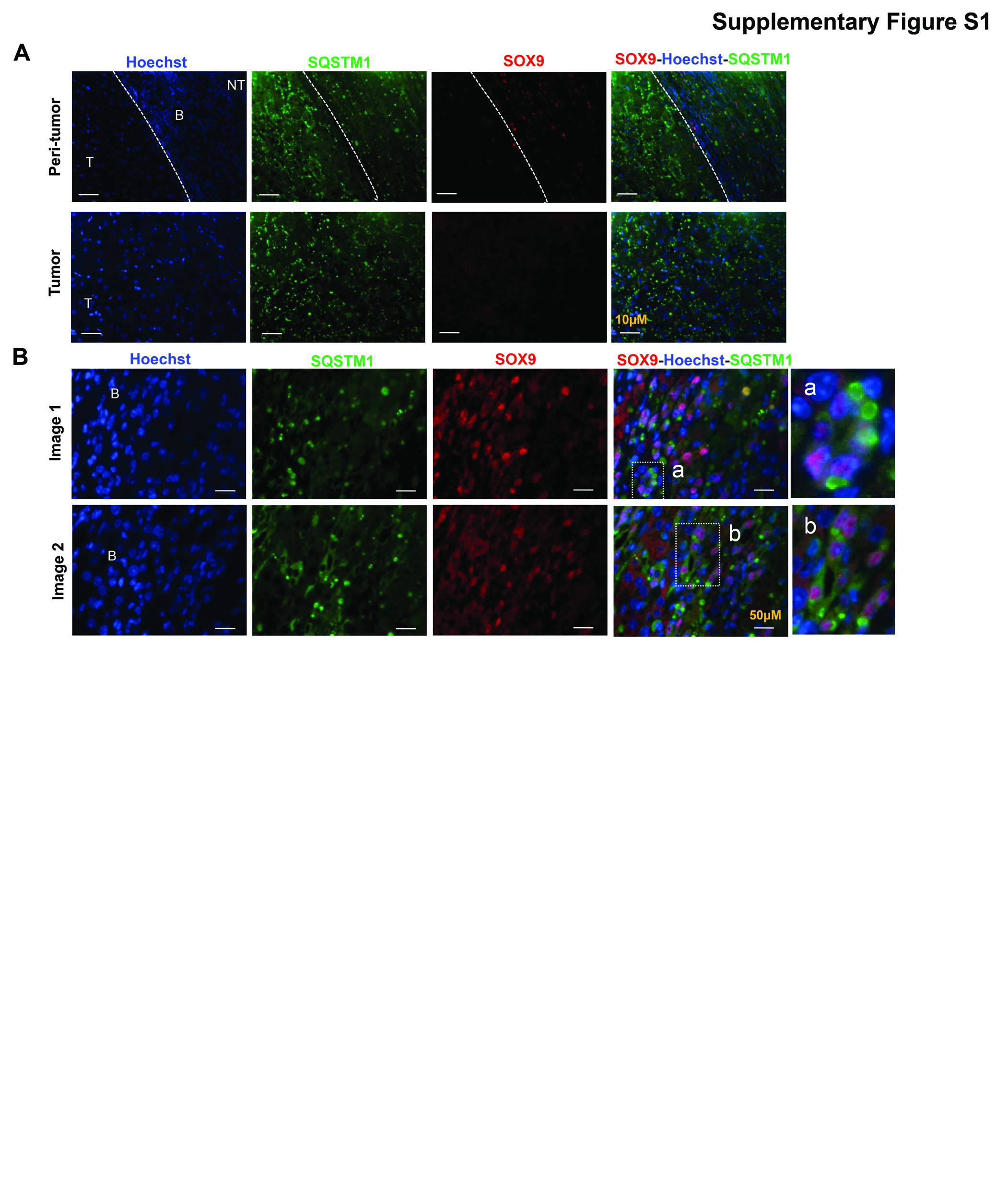

Supplement: Supplementary file 1 — Supplementary Figure-S1 [file 41419_2020_2536_MOESM1_ESM.tif]

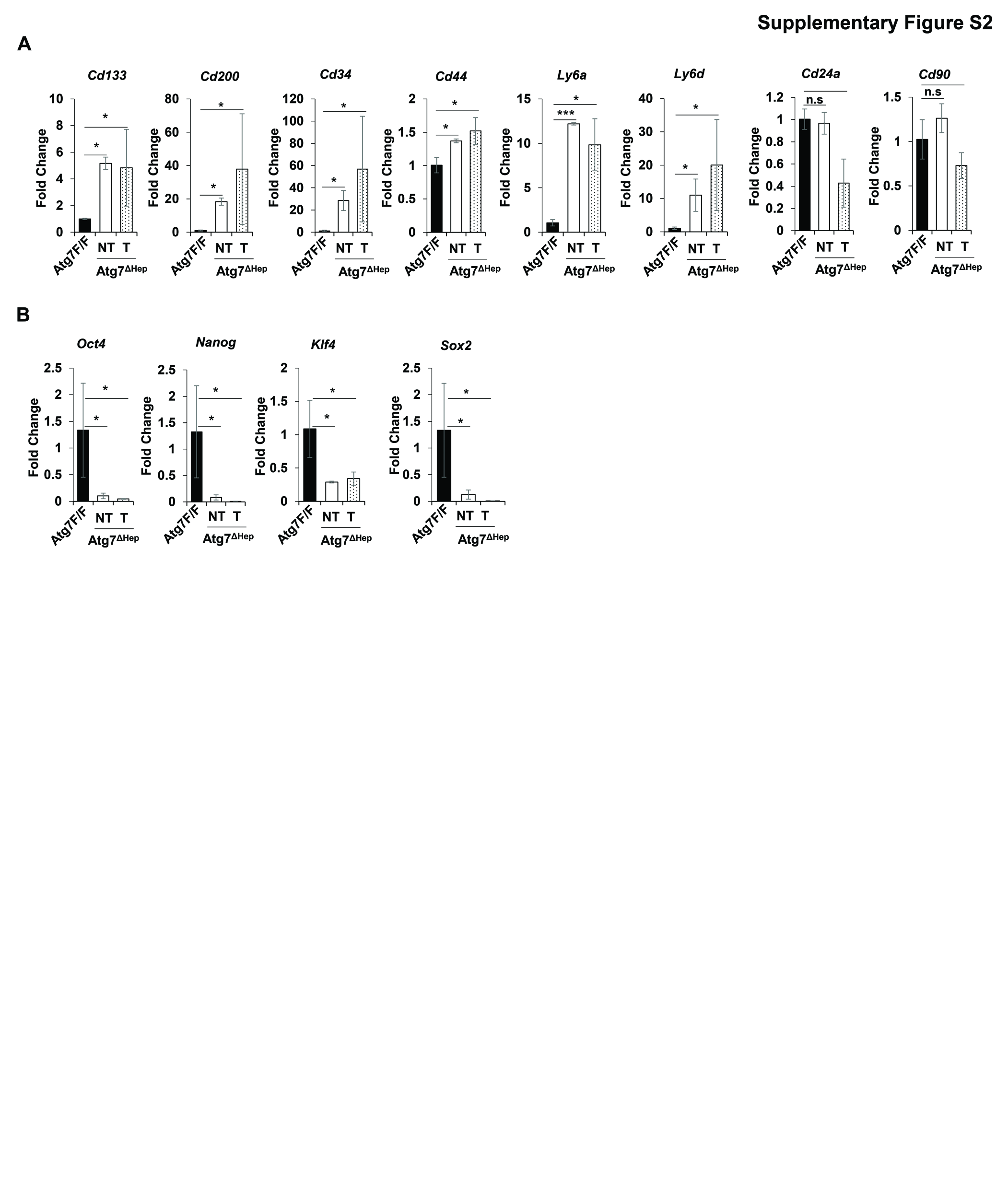

Supplement: Supplementary file 2 — Supplementary Figure-S2 [file 41419_2020_2536_MOESM2_ESM.tif]

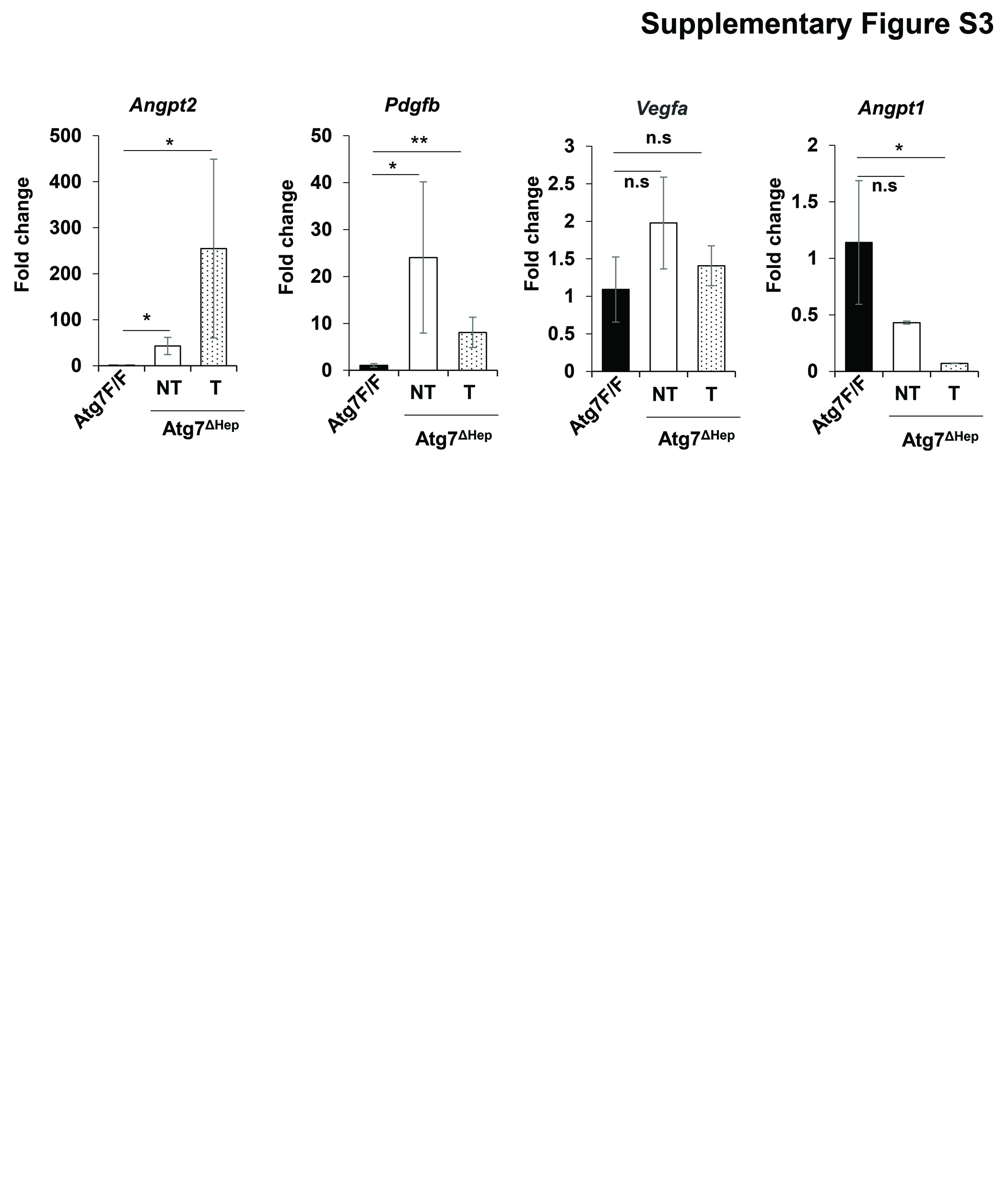

Supplement: Supplementary file 3 — Supplementary Figure-S3 [file 41419_2020_2536_MOESM3_ESM.tif]

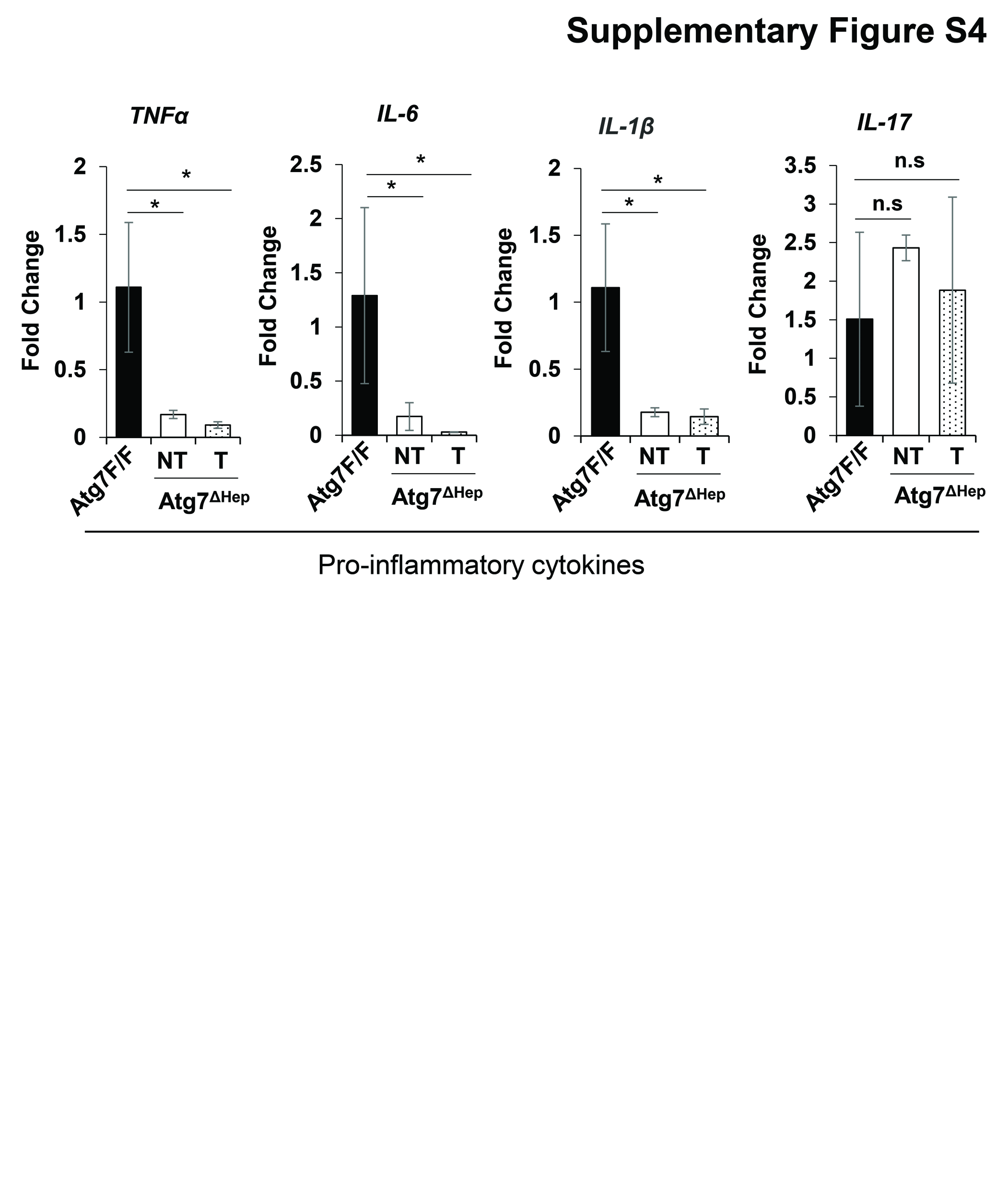

Supplement: Supplementary file 4 — Supplementary Figure-S4 [file 41419_2020_2536_MOESM4_ESM.tif]

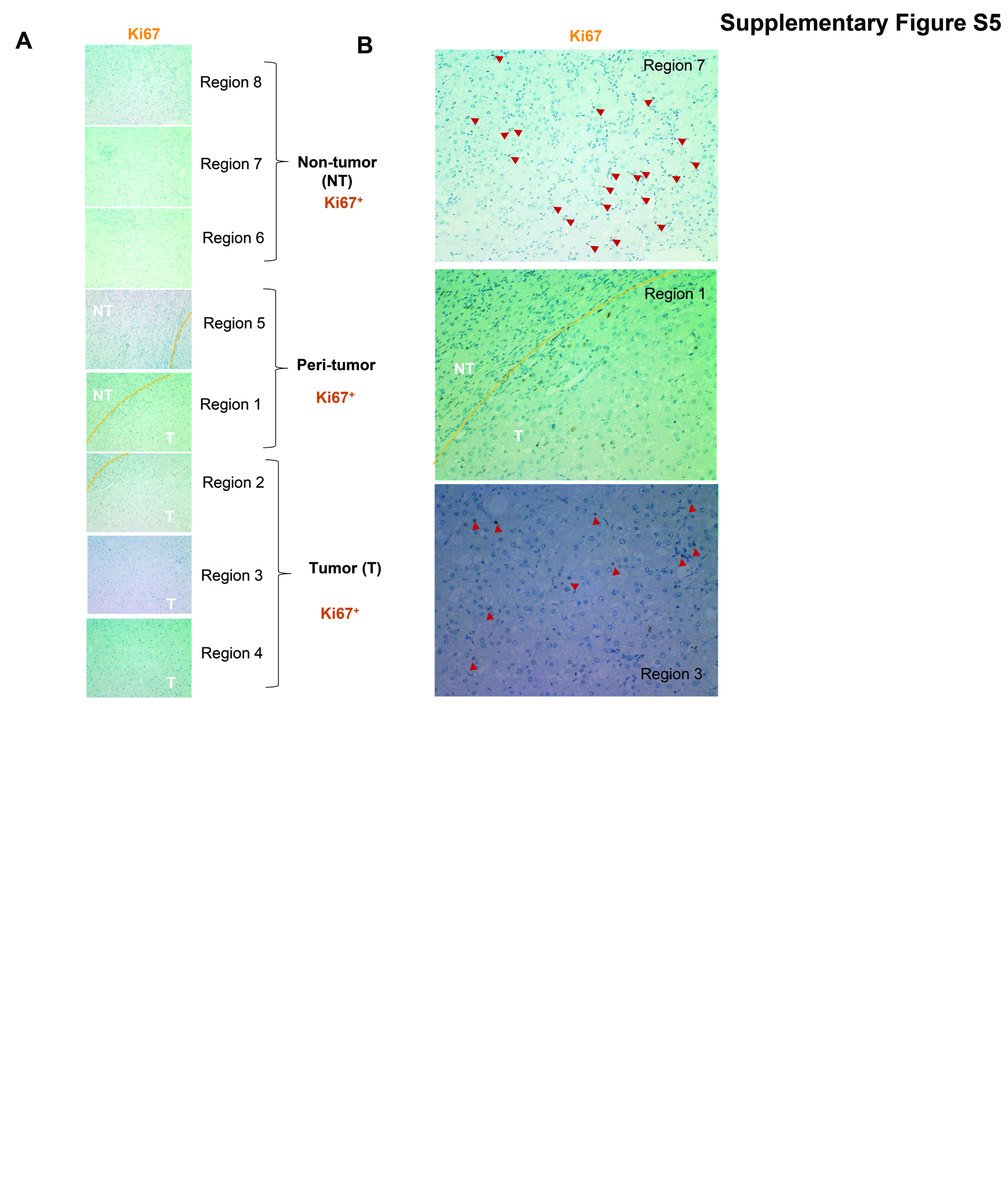

Supplement: Supplementary file 5 — Supplementary Figure-S5 [file 41419_2020_2536_MOESM5_ESM.tif]

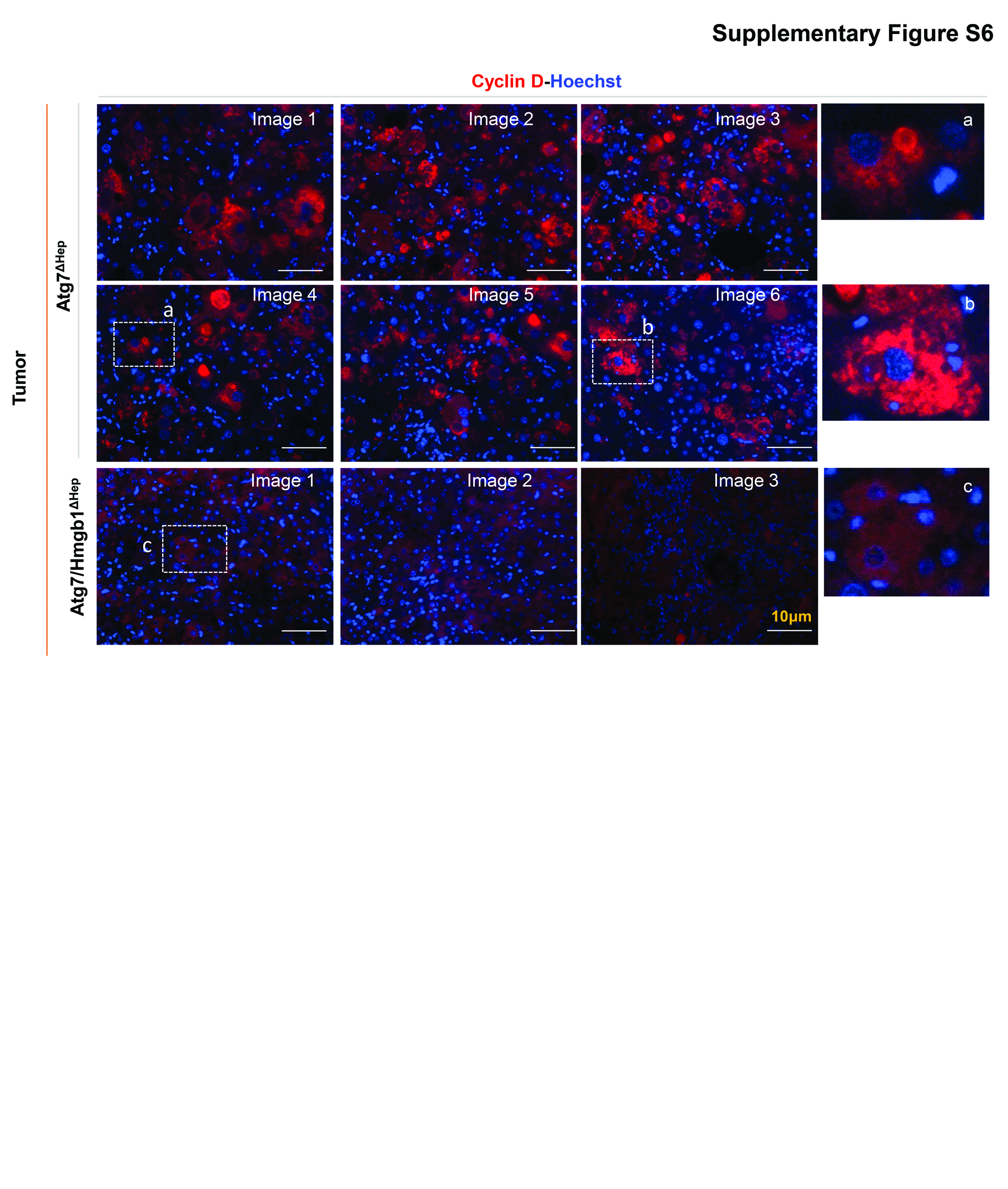

Supplement: Supplementary file 6 — Supplementary Figure-S6 [file 41419_2020_2536_MOESM6_ESM.tif]
