# Supplementary material for: The HMGB1-RAGE axis modulates the growth of autophagy-deficient hepatic tumors
Source: Cell Death Dis. 2020 May 7;11(5):333. doi: 10.1038/s41419-020-2536-7 (PMC7206028; doi:10.1038/s41419-020-2536-7)
Supplement: Supplementary file 8 — Supplementary Figure legends [file 41419_2020_2536_MOESM8_ESM.docx]

**Supplementary Figure legends**

**Supplementary Figure S1.** SOX9 positive hepatic progenitor cells are localized outside the tumor regions. Liver sections from 12-month old mice of *Atg7^ΔHep^* genotype were co-immunostained with anti-SQSTM1 and anti-SOX9. Images were taken around the peri-tumor and tumor region (**A**). Additional higher magnification images in the peri-tumor regions (**B**) were taken in order to show the SQSTM1 staining in SOX9 positive hepatic progenitor cells. The framed area is enlarged and shown in separate panels. White dotted lines indicate the tumor border. NT, non-tumor liver, B, peri-tumor, T, tumor.

**Supplementary Figure S2.** Elevation of Cancer stem cell-associated genes in non-tumor and tumor samples of autophagy-deficient livers. The hepatic mRNA expression level of CSCs marker genes (*Cd133/Prom1*, *Cd200/Ox-2*,*Cd34*, *Cd44*, *Ly6a/Sca-1*, *Ly6d, Cd24A/Has*, *Cd90/Thy1)* (**A**), and Stemness genes (*Oct4*, *Nanog*, *Klf4*, and *Sox2*) (**B**) in 15-month old *Atg7F/F*, and *Atg7^ΔHep^* mice were determined by real-time PCR. NT. non-tumor, T, tumor. Data are reported as mean± SE, * *P*<0.05, *** *P*<0.00, n.s.: no significance; n=3 mice per group.

**Supplementary Figure S3.** Angiogenic factors are altered in the tumor-bearing autophagy-deficient livers**.** The hepatic mRNA expression level of angiogenic factors (*Angpt2*, *Pdfgb Vegfra*, and *Angpt1)* in 15-month old *Atg7F/F* and *Atg7^ΔHep^* mice were determined by real-time PCR. NT, non-tumor, T, tumor. Data are reported as mean± SE, * *P*<0.05, ** *P*<0.01, n.s., no significance; n=3 mice per group.

**Supplementary Figure S4.** Expressional analysis of proinflammatory cytokine genes in tumor and non-tumor samples of autophagy-deficient livers. The hepatic mRNA expression level of inflammatory cytokines (*TNFα*, *IL-6*, *IL-1β* and, *IL-17)* in 15-month old *Atg7F/F* and *Atg7^ΔHep^* mice were determined by real-time PCR. NT, non-tumor, T, tumor. Data are reported as mean± SE,* *P*<0.05, n.s., no significance; n=3 mice per group.

**Supplementary Figure S5.** The autophagy-deficient tumors are proliferative. Liver sections from 12-month old mice of *Atg7^ΔHep^* genotype were subjected to immunohistochemistry for Ki67 (**A**) (original magnification, X200). Dotted lines indicate the tumor border. (**B**) Enlarged images of Region 1 (peri-tumor), Region 3 (tumor) and Region 7 (non-tumor) are shown in separate panels. Red arrow indicated Ki67 positive proliferating hepatocytes. NT, non-tumor, T, tumor.

**Supplementary Figure S6.** Cyclin D expression in autophagy-deficient livers. Livers of 15-month old mice of *Atg7^ΔHep^* and *Atg7/Hmgb1^ΔHep^* genotypes were immunostained with anti-Cyclin D. Several images were taken focusing in the tumor region. The framed area is enlarged and shown in separate panels. White dotted lines indicate the tumor border.

**Supplementary Figure S7.** Loss of *Hmgb1* activates AKT and JNK signaling but does not affect mTORC1, MAPK/ERK and STAT signaling in the autophagy-deficient livers. (**A**-**B**) Immunoblot analysis of AKT pathway, mTORC1 pathway, MAPK/ERK pathway (A), JNK pathway and , STAT pathway (B) related proteins in the tumor or non-tumor sample of 15-month old *Atg7^ΔHep^* and, *Atg7/Hmgb1^ΔHep^* mice. NT, non-tumor, T, tumor.

**Supplementary Tables**

1. **Supplementary Table S1.** List of overlapped genes that are upregulated or downregulated in tumors of both *Atg7^ΔHep^* and *Atg7/Hmgb1^ΔHep^* liver.
2. **Supplementary Table S2.**  List of upregulated genes in tumors of *Atg7^ΔHep^* liver.
3. **Supplementary Table S3.**  List of upregulated genes in tumors of *Atg7/Hmgb1^ΔHep^* liver.
4. **Supplementary Table S4.**  List of downregulated genes in tumors of *Atg7^ΔHep^* liver.
5. **Supplementary Table S5.**  List of downregulated genes in tumors of *Atg7/Hmgb1^ΔHep^* liver.
6. **Supplementary Table S6.** Summary of distribution of hepatic cells in non-tumor, peri-tumor and tumor tissues of the autophagy-deficient liver.
7. **Supplementary Table S7.** List of Primers used for qPCR.
8. **Supplementary Table S8.** List of Antibodies used for immunostaining and western blot.
